# Structural Basis for a Cork-Up Mechanism of the Intra-Molecular Mesaconyl-CoA Transferase

**DOI:** 10.1021/acs.biochem.2c00532

**Published:** 2022-12-19

**Authors:** Pascal Pfister, Jan Zarzycki, Tobias J. Erb

**Affiliations:** † Department of Biochemistry & Synthetic Metabolism, Max Planck Institute for Terrestrial Microbiology, Karl-von-Frisch Straße 10, 35043 Marburg, Germany; ‡ SYNMIKRO Center for Synthetic Microbiology, Karl-von-Frisch Straße 14, 35032 Marburg, Germany

## Abstract

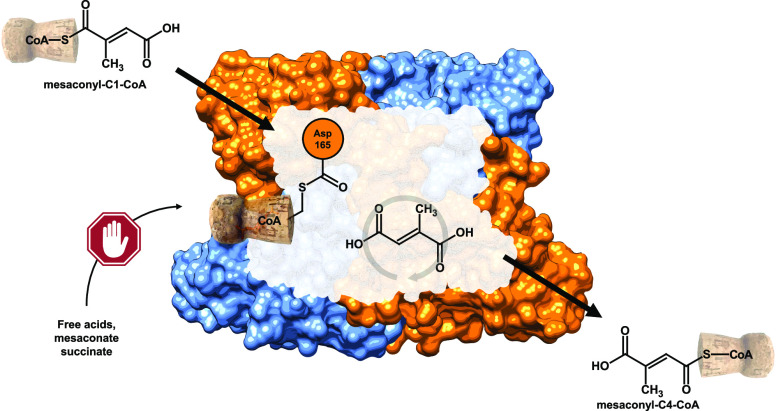

Mesaconyl-CoA transferase (Mct) is one of the key enzymes of the 3-hydroxypropionate (3HP) bi-cycle for autotrophic CO_2_ fixation. Mct is a family III/Frc family CoA transferase that catalyzes an unprecedented intra-molecular CoA transfer from the C1-carboxyl group to the C4-carboxyl group of mesaconate at catalytic efficiencies >10^6^ M^–1^ s^–1^. Here, we show that the reaction of Mct proceeds without any significant release of free CoA or the transfer to external acceptor acids. Mct catalyzes intra-molecular CoA transfers at catalytic efficiencies that are at least more than 6 orders of magnitude higher compared to inter-molecular CoA transfers, demonstrating that the enzyme exhibits exquisite control over its reaction. To understand the molecular basis of the intra-molecular CoA transfer in Mct, we solved crystal structures of the enzyme from *Chloroflexus aurantiacus* in its apo form, as well as in complex with mesaconyl-CoA and several covalently enzyme-bound intermediates of CoA and mesaconate at the catalytically active residue Asp165. Based on these structures, we propose a reaction mechanism for Mct that is similar to inter-molecular family III/Frc family CoA transferases. However, in contrast to the latter that undergo opening and closing cycles during the reaction to exchange substrates, the central cavity of Mct remains sealed (“corked-up”) by the CoA moiety, strongly favoring the intra-molecular CoA transfer between the C1 and the C4 position of mesaconate.

## Introduction

**Figure 1:**
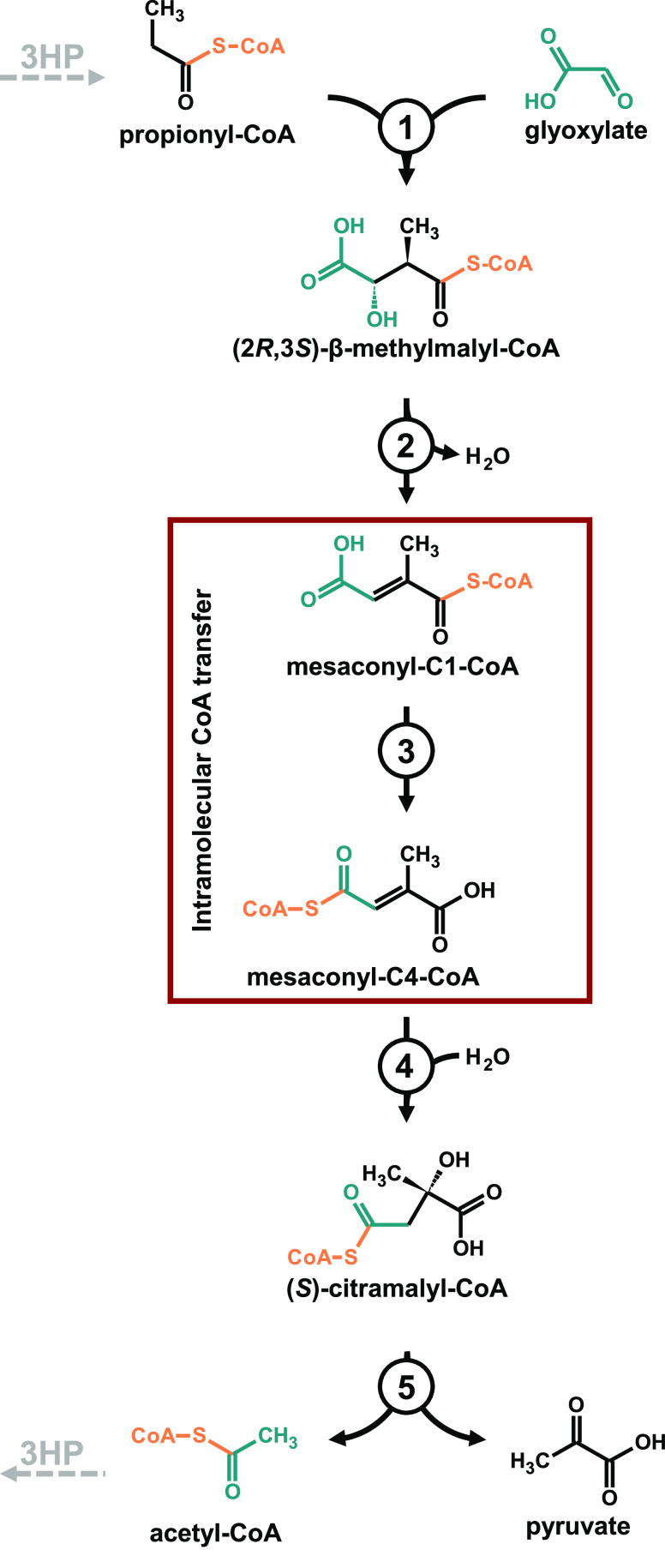
Reaction sequence of the 3HP bi-cycle^1^ involving Mct: (*S*)-malyl-CoA/(2*R*,3*S*)-β-methylmalyl-CoA/(3*S*)-citramalyl-CoA lyase (1 and 5), mesaconyl-C1-CoA hydratase (2), mesaconyl-C1:C4-CoA CoA transferase (3), and mesaconyl-C4-CoA hydratase (4). Metabolic connection to the 3HP bi-cycle is indicated in dashed lines. The atoms originating from glyoxylate are colored in teal. The CoA moiety is colored in orange.

The thermophilic green non-sulfur bacterium *Chloroflexus aurantiacus* uses the 3HP bi-cycle for autotrophic CO_2_ fixation.^1−3^ In the first part of the 3HP bi-cycle, CO_2_ is captured via two biotin-dependent carboxylases yielding glyoxylate as the primary CO_2_-fixation product. In the second part of the 3HP bi-cycle, glyoxylate is condensed with propionyl-CoA into (2*R*,3*S*)-β-methylmalyl-CoA.^4^ Methylmalyl-CoA is rearranged and converted into acetyl-CoA and pyruvate, the final CO_2_-fixation product.^1^ The rearrangement sequence of the second cycle starts via dehydration of methylmalyl-CoA into mesaconyl-C1-CoA (2-methylfumaryl-CoA).^5^ The CoA moiety of mesaconyl-C1-CoA is then transferred from the C1- to the C4-carboxyl group by Mct, resulting in mesaconyl-C4-CoA (3-methylfumaryl-CoA). Mesaconyl-C4-CoA is further converted into (3*S*)-citramalyl-CoA, which is ultimately cleaved into acetyl-CoA and pyruvate^1^ (Figure 1).

The Mct reaction is a key reaction in the 3HP bi-cycle. It conserves the energy-rich CoA-ester bond during C1-/C4-transfer without the release of mesaconate or the transfer of CoA onto other acceptors, which would result in a loss of intermediates and require additional ATP for (re-)activation of the free mesaconate. Overall, this makes the intra-molecular C1-/C4-CoA transfer by Mct an elegant and energetically highly efficient solution.

CoA transferases have been traditionally categorized into three different families, although recent phylogenetic analysis indicates that the evolutionary history of family I and II CoA transferases is more complex and that CoA transferases fall into six different monophyletic groups^6^ (see Table S1 for Pfams). “Family II” members (i.e., members of the CitF and MdcA families) are enzyme complexes that naturally use acyl-carrier proteins during catalysis but are also able to accept CoA esters as substrates *in vitro*.^7−9^ In contrast, “family I” members (i.e., members of the Cat1, OXCT1, and Gct families) and family III members (i.e., members of the Frc family) are lone-standing enzymes that typically catalyze the inter-molecular CoA transfer between a CoA donor and an acceptor acid in a similar fashion.^10−13^ The initial step in these enzymatic reactions is the nucleophilic attack of an active site, glutamate (“family I” members) or aspartate (family III/Frc family members), on the donor CoA ester, resulting in an acyl-enzyme anhydride and free CoAS^–^. The CoAS^–^ subsequently attacks the acyl-enzyme anhydride, releasing the donor acid and yielding a γ-glutamyl-bound (“family I”) or β-aspartyl-bound (family III/Frc family) enzyme-CoA thioester intermediate. The acceptor acid attacks the enzyme-CoA thioester to release CoAS^–^ and forms another acyl-enzyme anhydride. In the last step, this anhydride is re-attacked by the CoAS^–^, releasing the new CoA thioester.^9,10,12,14,15^

The catalytic mechanisms of “family I” and family III/Frc family CoA transferases follow similar principles. However, while “family I” transferases use a classical ping-pong mechanism,^11−13^ family III/Frc family enzymes show a modified mechanism, in which access of small acceptor acids to the active site may be gated either through a flexible glycine loop^10,16−20^ or even larger domain movements as observed for crotonobetainyl-CoA:carnitine CoA transferase (CaiB).^16^ The glycine-rich loop presumably opens and closes during the catalytic cycle to allow access of the acceptor acid upon formation of the β-aspartyl-CoA intermediate–with the donor acid still present at the active site. After CoA transfer, the newly formed acceptor acid-CoA thioester and the then free donor acid are released. Crystallographic evidence for these enzyme-bound intermediate states was presented for the formyl-CoA transferase (Frc) of *Oxalobacter formigenes*.^10^

While Mct falls within canonical family III/Frc family CoA transferases, the enzyme catalyzes an unprecedented intra-molecular CoA transfer, in which the acceptor acid (i.e., the second carboxylic group of mesaconate) is already part of the CoA donor. Since there is no need to introduce an additional substrate during the catalytic cycle, it has been speculated that the active site stays fully closed during catalysis.^1,21^ This hypothesis is consistent with the observation that small inactivating molecules that could react with the acyl-enzyme anhydride intermediate, such as hydroxylamine or borohydride, had little or even no effect on Mct activity.^1^ However, this also means that mesaconate would need to re-orient within the active site of Mct to enable CoA transfer from C1 to C4. Because of these proposed major differences to the catalytic cycle of inter-molecular CoA transferases, the mechanism of intra-molecular CoA transfer by Mct remained elusive.

Recently, the structure of the Mct homologue from *Roseiflexus castenholzii* (PDB 7XKG) was reported in its apo form.^21^ This structure showed that a flexible glycine-rich loop that supposedly gates catalysis in some other inter-molecular family III/Frc family CoA transferases^10,19^ is absent in Mct, indicating that the reaction may proceed differently in the intra-molecular CoA transferases. Based on the structure of the apoenzyme, molecular dynamics simulations with mesaconyl-C1- and C4-CoA were performed^21^ and a mechanism for the intra-molecular CoA transfer of Mct was proposed, which differed from the canonical family III/Frc family CoA transferases. Notably, a direct, water-assisted attack of the free CoAS^–^ onto the free carboxy group of mesaconate has been postulated.^21^ However, this mechanism seems biochemically infeasible and support for this mechanism is lacking.

Here, we sought to further biochemically and structurally characterize Mct from *C. aurantiacus* to better understand the molecular basis of catalysis. We show that Mct is virtually an exclusive intra-molecular CoA transferase and provide atomic-resolution crystal structures of the enzyme with different bound intermediates. Based on this data, we propose a mechanism for Mct that is similar to those of inter-molecular family III/Frc family transferases with the enzyme’s active site being “corked-up” by the substrate’s CoA moiety. This active site sealing likely favors the intra-molecular CoA transfer over inter-molecular CoA transfer in Mct by several orders of magnitude, resulting in a highly selective enzyme.

## Materials and Methods

### Synthesis of CoA Thioesters

#### Synthesis of Mesaconyl-C1- and Mesaconyl-C4-CoA

**Figure 2:**
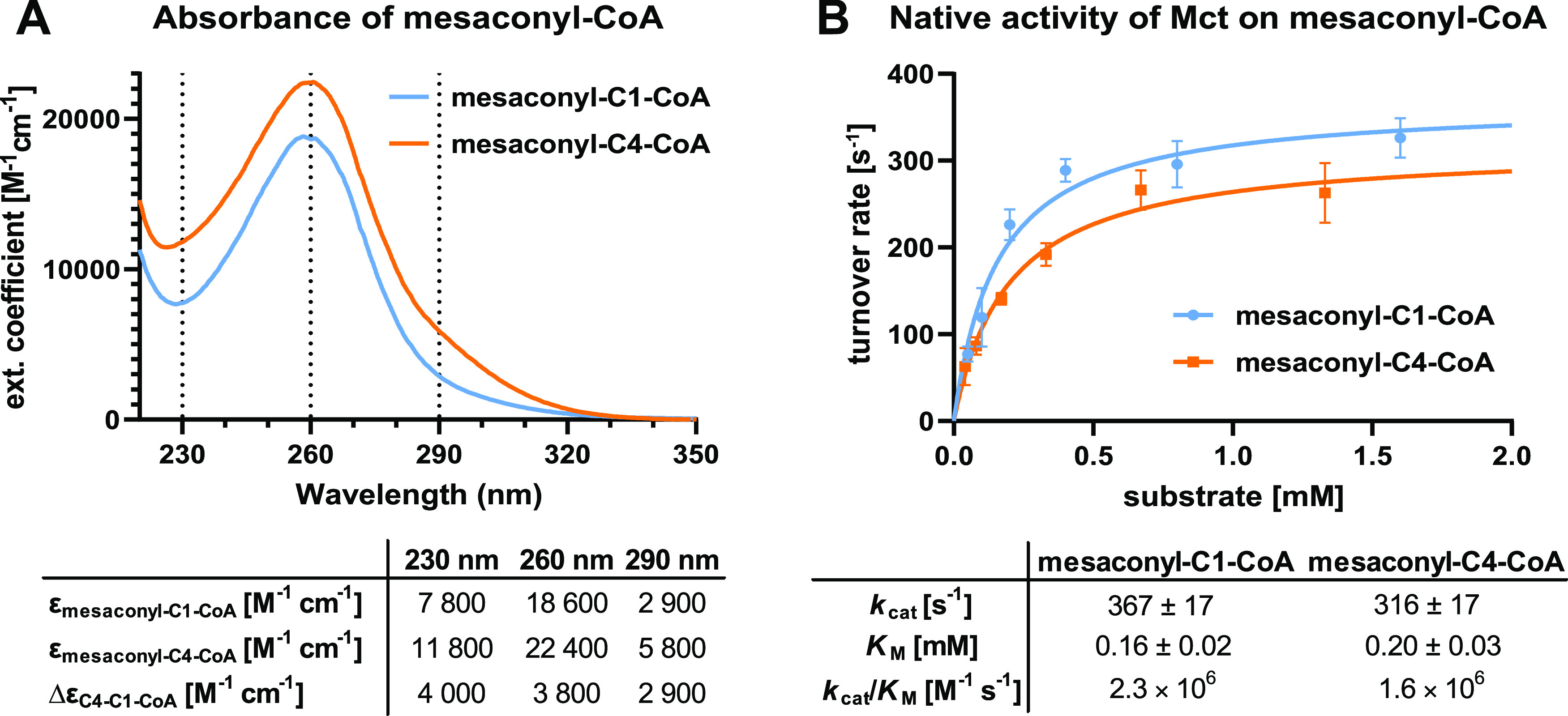
Spectrophotometric data for mesaconyl-CoA thioesters and the CoA transferase reaction. (A) UV spectra of mesaconyl-C1-CoA (blue) and mesaconyl-C4-CoA (orange). Extinction coefficients at 230, 260, and 290 nm for each substrate are given below The difference in absorbance at 290 nm (Δε_C4-C1-CoA_) was used for photometric activity assays. (B) Michaelis–Menten plot for Mct activity with mesaconyl-C1-CoA (blue) and mesaconyl-C4-CoA (orange), respectively. Kinetic values for both substrates are given in the bottom table. SD values are indicated.

First, 0.5 M mesaconic acid (116 mg) was dissolved in 2 mL of diethylether on ice. Then, 80 μL water-free pyridine and 94 μL of ice-cold ethyl chloroformate were added under constant stirring. After 15 min, the supernatant containing mesaconic anhydride was slowly added to a CoA solution (2.5 mM CoA, 25 mM NaHCO_3_). After 30 min of constant stirring on ice, pH was adjusted to pH of 3.0 with HCl.^22^ Free CoA, mesaconyl-C1-CoA, and mesaconyl-C4-CoA were separated by HPLC (Agilent 1260 Infinity HPLC) with a Gemini 10 μm NX-C18 110 Å column (Phenomenex) in a gradient from 14 to 50% methanol in buffer (25 mM NH_4_COOH/HCOOH, pH 4.2) over 10 min at a flow rate of 25 mL/min. Mesaconyl-C1- and C4-CoA could be differentiated by their UV spectra (Figure 2) and retention times. The retention times for CoA, mesaconyl-C1-CoA, and mesaconyl-C4-CoA were 1.9, 3.9, and 4.8 min, respectively. Peak fractions were pooled, frozen in liquid nitrogen, and subsequently lyophilized. The resulting powder was stored at −20 °C and solved in ddH_2_O before use. Purity was confirmed by HPLC–MS. Both CoA thioesters were >99% pure. They did not show any cross-contamination with the respective other mesaconyl-CoA derivative (Figure S1) or with free CoA, as judged by Ellman’s reagent.

#### Synthesis of Other CoA Esters

All other CoA thioesters were synthesized and purified according to previously established protocols.^23,24^

### Gene Expression and Protein Purification

The expression plasmid pMCTCa_JZ05 encoding a His-tagged Mct from *C. aurantiacus*(1) was used for protein production. The plasmid was transformed into *Escherichia coli* BL21 DE3, grown in 2 L Terrific Broth^25^ for 24 h at 25 °C without induction. The cells were harvested at 4 °C and 8000*g* and re-suspended in a threefold volume (3 mL per 1 g of cells) of loading buffer (50 mM MOPS/KOH pH 7.8, 150 mM NaCl, 75 mM imidazole). The cells were lysed using an LM10 microfluidizer (H10Z chamber, Microfluidics, Westwood, MA) at 18 000 psi. The lysate was heat-precipitated at 70 °C for 20 min and kept on ice for downstream purification. The unwanted denatured proteins were removed by centrifugation at 4 °C and 100 000*g* for 1 h. The cell extract was filtered (0.4 μm syringe filter), and Mct was purified by nickel affinity chromatography (elution buffer 50 mM MOPS/KOH, pH 7.8, 150 mM NaCl, 500 mM imidazole) using a 1 mL HisTrap FF column (Cytiva, Freiburg, Germany). Afterward, the eluate was desalted in low-salt buffer (50 mM MOPS/KOH, pH 7.8, 50 mM NaCl) and further purified by anion exchange (Q-HP 16/10 column, Cytiva, Freiburg, Germany) using a gradient with high-salt buffer (50 mM MOPS/KOH, pH 7.8, 300 mM NaCl) over 20 min. The enzyme eluted between NaCl concentrations of 100 and 150 mM. The purity of the enzyme was checked by SDS-PAGE at each purification step, and Mct was concentrated by centrifugal filters (Amicon by Merck, Darmstadt, Germany) with a 30 kDa cutoff. Protein concentrations were determined using a NanoDrop (ThermoFisher Scientific) and applying a calculated molar extinction coefficient of 49 000 M^–1^ cm^–1^ coefficient at 280 nm.

### Determination of the Extinction Coefficient of Mesaconyl-CoA Derivatives

UV spectra (220–350 nm) of both HPLC-purified mesaconyl-CoA forms were recorded via spectrophotometer (Cary 60, Agilent). CoA thioester concentrations were measured by depletion in a coupled NADPH-dependent assay^26^ by reduction via succinate-semialdehyde dehydrogenase (SucD, EC:1.2.1.76) spectrophotometrically^27^ (Cary 60, Agilent) in a 1 cm quartz cuvette (300 μL assay volume; 200 mM HEPES, pH 7.5, 70 nM SucD, 0.7 mM NADPH and about 0.25 mM mesaconyl-CoA) at 365 nm and 37 °C.

### Determination of Enzymatic Activity

#### Spectrophotometric Assay

To examine the activity of the intra-molecular CoA transfer of Mct, a spectrophotometric assay was used. Mesaconyl-C4-CoA has a higher extinction coefficient at 290 nm (ε_290 nm_ = 5800 M^–1^ cm^–1^) than mesaconyl-C1-CoA (ε_290 nm_ = 2900 M^–1^ cm^–1^). Therefore, the conversion of mesaconyl-C1-CoA was measured by the increase in absorbance at 290 nm (Δε_290 nm_ = 2900 M^–1^ cm^–1^). To conduct the measurements, 150 μL assay volume (200 mM HEPES/KOH, pH_25 °C_ 8.0, 22 nM Mct, and varying concentrations of mesaconyl-CoA) was incubated at 55 °C, and change in absorbance at 290 nm was monitored over time in a 3 mm quartz cuvette. The reaction was started with the substrate (ranging from 50 to 1600 μM for mesaconyl-C1-CoA and 40 to 1300 μM for mesaconyl-C4-CoA).

#### HPLC–MS-Based Assay

To test for alternative CoA acceptors, Mct was preincubated in reaction buffer (200 mM HEPES, pH 7.5) and supplemented with 20 mM of the corresponding carboxylic acid. After 5 min of preincubation, the reaction was started by the addition of 1 mM mesaconyl-C1-CoA. Samples were taken after 0 and 20 min and stopped on ice by the addition of HCl to a final concentration of 100 mM. The precipitated enzyme was removed by centrifugation (4 °C and 17 000*g*), and the supernatants were analyzed by HPLC-MS and for the presence of alternative CoA thioesters.

To evaluate and quantify the kinetics of succinate as acceptor acids, an enzyme assay was performed (55 μL; 200 mM HEPES/KOH, pH_25 °C_ 8.0, 6 μM Mct, 1 mM mesaconyl-C4-CoA, and varying concentrations of succinate). The reaction was started with the addition of succinate and incubated for 20 min at 55 °C. At 0, 1, and 20 min, a sample of 5 μL was taken and quenched in 45 μL of formic acid. The precipitated enzyme was removed by centrifugation (4 °C and 17 000*g*), and the supernatants were analyzed by HPLC-MS for the presence of succinyl-CoA.

Determination of CoA thioesters was performed using a HiRes-LC-MS. The chromatographic separation was performed on a Thermo Scientific Vanquish HPLC system using a Kinetex Evo C18 column (150 × 2.1 mm^2^, 100 A, 1.7 μm, Phenomenex) equipped with a 20 × 2.1 mm^2^ guard column of similar specificity at a constant eluent flow rate of 0.25 mL/min and a column temperature of 25 °C with eluent A being 50 mM ammonium formate at a pH of 8.1 water and eluent B being MeOH (Honeywell). The injection volume was 1 μL. The elution profile consisted of the following steps and linear gradients: 0–2 min constant at 0% B; 2–10 min from 0 to 80% B; 10–12 min constant at 80% B; 12–12.1 min from 80 to 0% B; and 12.1–15 min constant at 0% B. A Thermo Scientific ID-X Orbitrap mass spectrometer was used in positive mode with an electrospray ionization source and the following conditions: ESI spray voltage 3500 V, sheath gas at 50 arbitrary units, auxiliary gas at 10 arbitrary units, sweep gas at 1 arbitrary unit, ion transfer tube temperature at 300 °C, and vaporizer temperature at 350 °C. Detection was performed in full-scan mode using the orbitrap mass analyzer at a mass resolution of 240 000 in the mass range 800–900 (*m*/*z*). Extracted ion chromatograms of the [M + H]^+^ forms were integrated using Tracefinder software (Thermo Scientific). Absolute concentrations for succinyl-CoA were calculated based on an external calibration curve.

### Crystallization of Mct X-ray Structure Determination

The purified protein solution was spotted in different concentrations (3, 6, and 8 mg/mL) on sitting-drop vapor-diffusion crystallization plates. First, 0.2 μL of each protein solution was mixed with 0.2 μL of crystallization condition. The drops were equilibrated against 30 μL of protein-free crystallization condition at 288 K. The resulting crystals of condition A (200 mM sodium chloride, 100 mM sodium potassium phosphate, pH 6.2, and 50% v/v poly(ethylene glycol) 200) appeared after 5 days. In wells containing condition B (35% 2-methyl-2,4-pentanediol and 100 mM sodium/potassium phosphate, pH 6.2) crystals appeared after 2 days and grew until the 5th day of incubation. The crystals in condition A were directly snap-frozen in liquid nitrogen, whereas the crystals of condition B were transferred into a drop containing higher concentrations of cryoprotectant and a mixture of both forms of mesaconyl-CoA (40% MPD, sodium/potassium phosphate, pH 6.2, 5 mM mesaconyl-CoA) for 2 min and were then frozen in liquid nitrogen. X-ray diffraction data were collected at the beamline ID29 of the European Synchrotron Radiation Facility (ESRF) and the beamline P14 of the Deutsches Elektronen-Synchrotron (DESY). The data sets were processed with the XDS software package.^28^ The structures were solved by molecular replacement using a polyalanine search model of the formyl-CoA:oxalate CoA transferase from *Acetobacter aceti* (PDB ID 3UBM).^29^ Molecular replacement was carried out using Phaser of the Phenix software package^30^ and refined with Phenix.Refine. Additional modeling, manual refining, and ligand fitting were done in COOT.^31^ Final positional and *B*-factor refinements, as well as water-picking for the structure, were performed using Phenix.Refine. The Mct structure models were deposited at the PDB in Europe under PDB IDs 8APR and 8APQ. Data collection and refinement statistics are provided in Table 1.

**Table 1:** Data and Refinement Statistics for the Mct Crystal Structures^a^

crystal	Mct – apo form	Mct with bound substrates
beamline	ESRF ID29, Grenoble, France	DESY P14, Hamburg, Germany
PDB ID	8APR	8APQ
ligands	3Cl^–^	mesaconyl-C1-CoA, mesaconate, CoA
wavelength	0.96862	0.97660
resolution range (Å)	29.2–2.1 (2.2–2.1)	29.7–2.5 (2.6–2.5)
space group	*C*121	*P*3_2_21
unit cell dimensions		
*a*, *b*, *c* (Å)	172.6, 103.5, 95.3	193.8, 193.8, 252.0
α, β, γ (deg)	90.0, 119.6, 90.0	90.0, 90.0, 120.0
total reflections	576 260 (51 434)	1 110 749 (111 537)
unique reflections	84 090 (8166)	189 902 (18 751)
multiplicity	6.9 (6.3)	5.8 (5.9)
completeness (%)	99.52 (96.95)	99.68 (99.38)
mean *I*/σ(*I*)	13.47 (2.28)	13.57 (2.68)
*R*_merge_	0.1011 (0.8707)	0.08911 (0.7038)
*R*_pim_	0.04167 (0.3762)	0.0401 (0.3118)
CC_1/2_	0.998 (0.864)	0.998 (0.804)
reflections used in refinement	84 021 (8139)	189 871 (18750)
*R*_work_	0.1808 (0.2690)	0.1850 (0.2386)
*R*_free_	0.2125 (0.3305)	0.1995 (0.2702)
number of non-hydrogen atoms	9886	20187
macromolecules	9354	18795
ligands	3	337
solvent	529	1055
protein residues	1212	2435
RMS (bonds)	0.007	0.002
RMS (angles)	0.86	0.49
Ramachandran		
favored (%)	98.09	97.36
allowed (%)	1.66	2.39
outliers (%)	0.25	0.25
rotamer outliers (%)	0.52	0.10
clashscore	1.60	1.07
average *B*-factor	41.76	50.62
macromolecules	41.62	50.30
ligands	35.78	69.70
solvent	44.32	50.19

a Statistics for the highest-resolution shell are in parentheses.

## Results

### Mct Is a Highly Efficient Intra-Molecular Mesaconyl-CoA Transferase

For the spectrophotometric kinetic characterization of Mct, we first synthesized and purified mesaconyl-C1-CoA and mesaconyl-C4-CoA. We revisited the UV spectra of both CoA thioesters to determine their exact extinction coefficients at 230, 260, and 290 nm. While the overall spectra of both compounds were similar, the C1 and C4 species showed distinct differences. Compared to mesaconyl-C1-CoA, the spectrum of mesaconyl-C4-CoA resembled more those of other α,β-unsaturated CoA esters like crotonyl- or acrylyl-CoA, exhibiting a higher overall extinction coefficient at 260 nm and a more pronounced shoulder in the region between 280 and 340 nm (Figure 2A).

We then used the difference in the extinction coefficients at 290 nm (Δε_290 nm_ = 2900 M^–1^ cm^–1^) to determine the catalytic properties of Mct from *C. aurantiacus* at the organism’s optimum growth temperature of 55 °C with mesaconyl-C1-CoA and mesaconyl-C4-CoA in a continuous photometric assay. Starting with either of the substrates, the enzyme showed remarkably high *V*_max_ values of 495 and 430 μmol min^–1^ mg^–1^ for mesaconyl-C1- and C4-CoA, corresponding to *k*_cat_ values of 370 and 320, respectively (Figure 2B). The *K*_M_ values for both CoA esters were 0.16 and 0.2 mM, resulting in catalytic efficiencies (*k*_cat_/*K*_M_) of 2.3 × 10^6^ and 1.6 × 10^6^ M^–1^ s^–1^ for mesaconyl-C1-CoA and mesaconyl-C4-CoA, respectively (Figure 2B). These kinetic parameters are in line with previously published values^1^ while also considering the revised extinction coefficients.

### Mct Strongly Discriminates against Other Substrates

**Figure 3:**
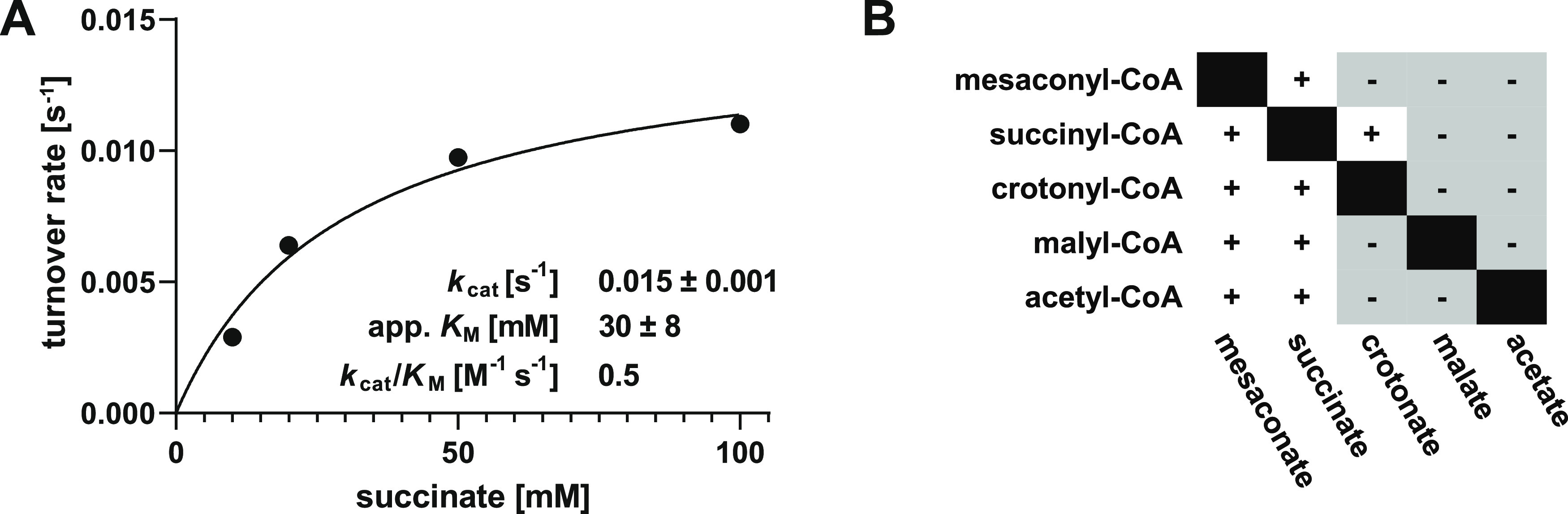
Testing externally provided acids and CoA esters for inter-molecular CoA transfer. (A) Kinetic parameters for mesaconyl-CoA:succinate transfer show very poor catalytic efficiency for succinate as an alternative CoA acceptor. The SD is indicated. (B) Testing different potential CoA donors and CoA acceptors shows that only succinate and mesaconate serve as CoA acceptors, whereas all tested CoA esters serve a as CoA donor for succinate and mesaconate. “+” indicates that CoA was transferred onto the respective acid, as confirmed by HPLC–MS. “–” indicates that the formation of a corresponding CoA thioester could not be detected by HPLC–MS.

Next, we wanted to assess Mct’s ability to use succinate as an alternative dicarboxylic acid-CoA acceptor when externally provided during catalysis with mesaconyl-CoA. We detected only a negligible side activity (i.e., formation of succinyl-CoA) with an extremely low catalytic efficiency for the CoA transfer onto succinate (*k*_cat_/*K*_M_ = 0.49 M^–1^ s^–1^), which is more than 6 orders of magnitude lower compared to the interconversion of the two different mesaconyl-CoA thioesters (see Figure 3A). This strong selectivity against free succinate was accompanied by a very high apparent *K*_M_ for this alternative substrate (>25 mM).

Having identified a very low, but detectable activity with succinate, we sought to test other central carbon metabolites as potential acceptor acids and several alternative acyl-CoA thioesters as potential CoA donors. To that end, we preincubated different carboxylic acids (mesaconate, succinate, malate, crotonate, and acetate) individually at concentrations of 20 mM for 5 min with Mct, before the reaction was started with 1 mM of either mesaconyl-, succinyl-, crotonyl-, or acetyl-CoA. In these assays, Mct also accepted crotonate as an alternative acceptor acid, when succinyl-CoA was provided as a CoA donor (Figure 3B). However, activity with crotonate as a CoA acceptor was comparable to or even lower than for succinate and several orders of magnitude lower than the intra-molecular reaction with mesaconyl-CoA alone. This demonstrated that Mct is able to efficiently discriminate against other carboxylic acids during catalysis.

When testing mesaconate as a CoA acceptor with different alternative CoA donors, we found that all of the tested CoA esters could in general serve as substrates (Figure 3B). However, mesaconyl-CoA formation only occurred when mesaconate was provided in nonphysiologically high concentrations (20 mM). These results are in line with previous data that reported a lack of detectable radioactive products when either ^14^C-labeled mesaconyl-CoA or ^14^C-labeled mesaconate was used with their respective unlabeled counterparts.^1^ We, therefore, reason that these (side) reactions are likely irrelevant under physiological conditions. Taken together, our data show that Mct can neither serve as a mesaconyl-CoA:carboxylic acid-CoA transferase nor possess a significant activity as acyl-CoA:mesaconate CoA transferase and therefore is a bona fide intra-molecular CoA transferase.

### Crystal Structure Reveals Snapshots of the Catalytic Cycle

**Figure 4:**
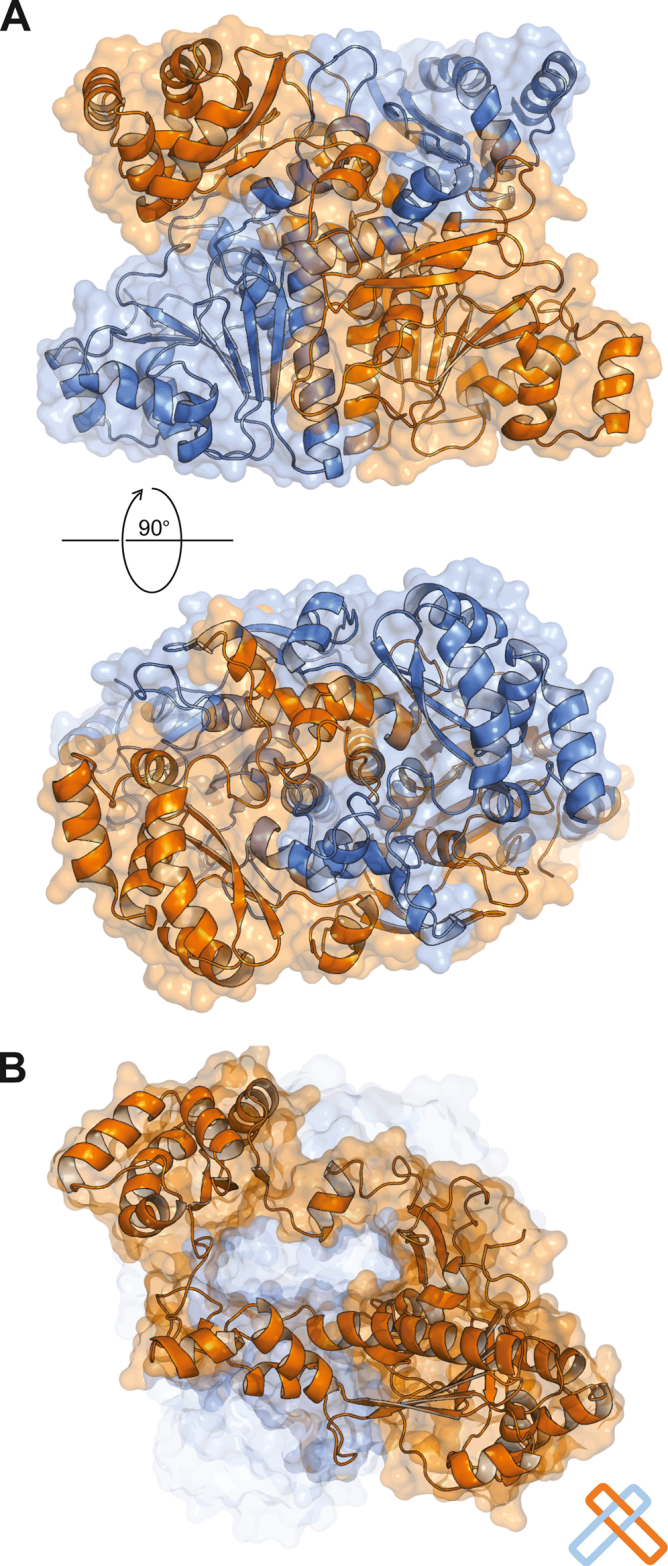
Active site of Mct. (A) Two subunits shown in orange and blue form an intertwined dimer depicted in cartoon and surface representations. (B) Family III CoA transferases form an interlocked dimer. Shown in surface representation are Mct subunit A in orange and subunit B in transparent blue. The polypeptide chains are interlocked and each is threaded through a hole in the neighboring subunit, as represented by the pictogram in the lower right corner.

Next, we became interested in understanding the structural determinants underlying substrate discrimination in Mct. We first solved the crystal structure of Mct from *C. aurantiacus* in its apo form without substrates at 2.1 Å resolution. Similar to the recently solved crystal structure of the homologue from *Roseiflexus*(21) and the other family III/Frc family CoA transferases,^10,16,18,29^ Mct of *C. aurantiacus* is an intertwined homodimer (Figure 4),^17^ where the polypeptide chains are threaded through a hole in the neighboring subunit (Figure 4B), respectively. A Rossman fold is formed between the C- and the N-termini of the enzyme. Residues Leu8 to Ala195 of the N-terminus form the essential part of the Rossman fold motif, followed by a loop that completely wraps around the adjacent subunit of the Mct dimer. This loop ends in a structure on the opposite side of the Rossman fold harboring three antiparallel β-strands and five short α-helices. Another loop reaches back to the described N-terminal structure, in which residues following Thr398 complete the Rossman fold.

**Figure 5:**
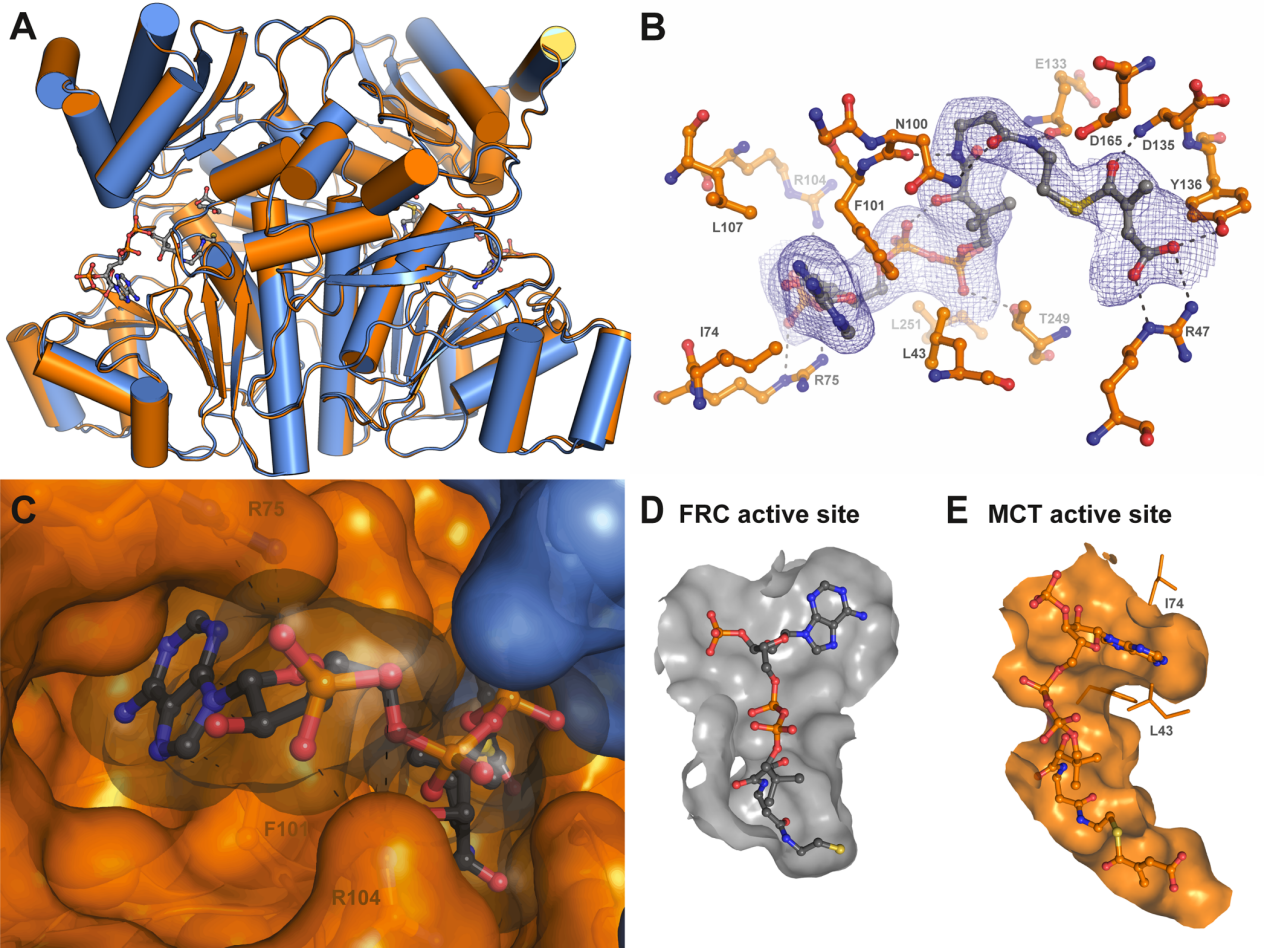
Structure of Mct. (A) Overlay of the apo form (blue) of Mct and the ligand-bound structure (orange) with an RMSD of 0.267 Å between 675 Cα-pairs. Bound substrates/intermediates are shown (gray) in ball and stick representation. (B) The mesh represents a simulated annealing omit map (*F*_o_–*F*_c_) at 2.0 σ, showing mesaconyl-C1-CoA bound to the active site. Polar interactions with surrounding residues are shown with dashed lines. (C) A transparent surface representation depicts the adenosyl moiety of CoA strongly coordinated at the mouth of the substrate tunnel by F101, R75, and R104, resulting in a cork-like sealing of the active site. (D) Shown is a slice through the active site cavity of the inter-molecular formyl-CoA transferase of *O. formigenes* (Frc) in complex with CoA (PDB 1P5R) and (E) the active site cavity of the intra-molecular Mct of C. aurantiacus in complex with mesaconyl-C1-CoA (gray) for comparison. Here L43 constricts the mouth of the active site cavity.

**Figure 6:**
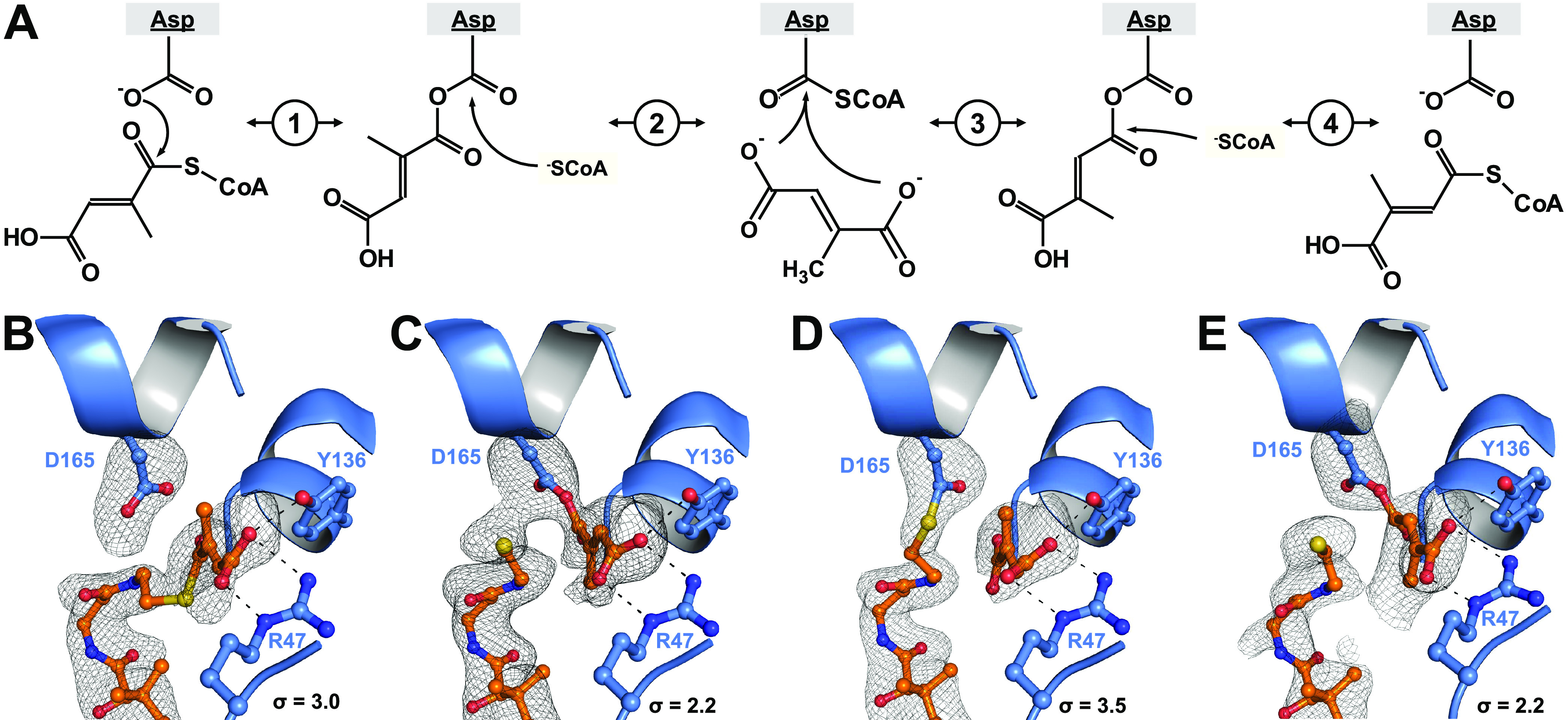
Proposed reaction mechanism of Mct. (A) An aspartate residue attacks the thioester bond of mesaconyl-C1-CoA (1). The free CoA attacks the mesaconyl-C1-aspartate and liberates mesaconate from the anhydride bond with Asp165 (2). Mesaconate flips around in the active site. The C4-carboxyl group attacks the afore-formed β-aspartyl-CoA (3). Finally, free CoA attacks the mesaconyl-C4-anhydride bond yielding mesaconyl-C4-CoA (4). (B–E) Crystallographically identified intermediates of the Mct reaction (PDB 8APQ). Simulated annealing omit maps (*F*_o_–*F*_c_) are shown as black mesh and the respective σ values are given. (B) Mesaconyl-C1-CoA was observed in chain A, (C) mesaconyl-C1-aspartate and CoA in chain E, (D) β-aspartyl-CoA and mesaconate in chain D, and (E) mesaconyl-C4-aspartate and CoA in chain F.

We also solved another structure of Mct under different crystallization conditions and with substrate soaking at 2.5 Å resolution. Under these conditions, we detected three dimers of Mct in the asymmetric unit (ASU). The structure of the soaked crystal showed additional electron densities at the six active sites representing different states of bound substrates and/or reaction intermediates. The active sites are located in cavities that are formed directly at the dimerization interfaces between the two subunits. They are located adjacent to the Rossman fold of each monomer and harbor the catalytically active Asp165 residue, which itself is part of the last helix of the Rossman fold. Although the electron densities at the active sites were slightly ambiguous, representing somewhat mixed states, we were able to model mesaconyl-C1-CoA (Figure 5), as well as Asp165–mesaconate anhydride intermediates with free CoA, and a β-aspartyl-CoA intermediate with free mesaconate into the different active sites present in the ASU, respectively (Figure 6).

In the active site with bound mesaconyl-C1-CoA, the mesaconyl moiety rests in close proximity to the catalytic Asp165. The terminal carboxy group of mesaconyl-CoA is coordinated by Arg47 and Tyr136 (see Figure 5B). Notably, Arg47 also occupies the corresponding space of the flexible glycine-rich loop that is found in some inter-molecular CoA transferases,^10,17^ preventing conformational changes, such as active-site opening or closing in Mct.

The phosphopantetheine arm of CoA is well coordinated along the active site tunnel of Mct, and the carbonyl–oxygen of the thioester bond engages in a hydrogen bridge with the peptide nitrogen of Asp135. The amide nitrogen, the amide oxygen of the β-alanine, and the cysteamine moiety of CoA are coordinated by the backbone oxygen of Glu133 and the side chain of Asn100, respectively. Arg75 and Arg104 coordinate with the phosphate of the adenosyl group. The adenine ring itself is wedged in between Phe101 and Ile74, engaging in a staggered π-stack with the phenylalanine (Figure 5B). Notably, the adenosyl group of CoA adopts a different, perpendicular (“kinked”) orientation to what is found in the other family III/Frc family enzymes (Figure 5D,E).^10,16−18^ In addition to this difference in adenine binding, Mct also harbors Leu43, which narrows the entrance to the active site of Mct substantially compared to inter-molecular CoA transferases (Figure 5D,E). A leucine or isoleucine residue in this position is conserved in all CoA transferases that catalyze intra-molecular CoA transfer, i.e., Mct from *C. aurantiacus*, *R. castenholzii*,^21^ Candidatus Accumulibacter phosphatis,^32^ and the γ1-endosymbiont of the gutless worm *Olavius algarvensis*.^33^ Overall, the tight binding of CoA along the substrate tunnel together with kinking of the adenine prevents trapped molecules from escaping and other molecules from entering the active site (Figure 5B–E), effectively sealing the catalytic site in a “cork-like” fashion.

Importantly, the CoA moiety also plugs those active sites, in which mesaconate is covalently bound to Asp165, indicating that the CoA moiety does not exchange during catalysis, which is consistent with our biochemical observations. This is also supported by previous experiments that concluded through radioactive labeling that no external mesaconate was involved in the reaction mechanism of Mct.^1^ Notably, electron densities in different active sites in the ASU allowed us to place the Asp165–mesaconate anhydride in the C1- as well as the C4-bound orientation (Figure 6). We did not observe any electron density that would accommodate an additional mesaconate molecule in any of the active sites. Altogether, these structures suggest that the intra-molecular transfer follows a similar mechanism as canonical inter-molecular, family III transferases that work with two distinct substrates—except that the substrate is not exchanged and may passively re-orient itself during catalysis. A small pocket around Arg47 appears large enough for mesaconate to change orientation randomly. Supporting this hypothesis, in one active site, we actually observed clear electron density for a β-aspartyl-CoA intermediate at Asp165 and a free mesaconate molecule in the aforementioned pocket (Figure 6D).

In summary, our structure with bound reaction intermediate states provides additional evidence and an explanation of how Mct catalyzes the intra-molecular CoA transfer favoring it over an inter-molecular transfer. Note that we did not observe any significant conformational changes between the apo form and the substrate-bound form of Mct (Figure 5A). This steric constraint of the apoenzyme together with the tight binding of CoA may effectively prevent access to the active site (Figure 5B–E), explaining how Mct is able to exclude other CoA acceptor carboxylic acids during the interconversion of the two forms of mesaconyl-CoA. Based on our crystal structures with different substrate-bound states (Figure 6B–E), Mct follows the canonical reaction mechanism proposed for the other family III CoA transferases (Figure 6A) with neither conformational changes nor exchanges of substrates taking place during the reaction.

## Discussion

Here, we biochemically and structurally characterized Mct, an unusual family III/Frc family CoA transferase that catalyzes an intra-molecular CoA transfer. Our structure with covalently enzyme-bound intermediates provides evidence that the enzyme follows the mechanism for inter-molecular family III/Frc family CoA transferases as proposed by Berthold et al.^10^ Based on our data, we suggest that upon mesaconyl-CoA entering the active site, Asp165 attacks the thioester bond, forming a mesaconyl-C1-aspartate anhydride and free CoA. The Asp165-bound mesaconate is displaced by an attack of the free CoA, resulting in a β-aspartyl-CoA, and releasing mesaconate into the active site cavity, where it can freely rotate within an extended pocket close to the catalytically active aspartate residue. At this step, any of the two carboxyl groups of mesaconate can attack the aspartyl-CoA, yielding either mesaconyl-C1-CoA or mesaconyl-C4-CoA.

The proposed reaction mechanism alone, however, does not explain why the reaction is specific for an intra-molecular transfer and how CoA transfer to other acceptor acids is prevented. The tight coordination of the CoA moiety effectively closes the active site and leads to an enclosed, “corked-up” reaction chamber, excluding diffusion of molecules in or out of the active site. Additionally, we did not observe any significant conformational changes in our two crystal structures, which could allow mesaconate to leave the active site or other acceptor acids to enter. While it could be in principle possible that an alternative acceptor acid may become trapped in the active site before mesaconyl-CoA or another CoA donor threads into the active site tunnel, this seems to be an unlikely event, as our assays with alternative acceptor acids showed that inter-molecular transfer is extremely rare and only takes place at very high, nonphysiologically relevant concentrations of these acids. Such a trapped acceptor molecule could interfere with the re-orientation of the mesaconate released from mesaconyl-CoA, rather resulting in the re-formation of the mesaconyl-CoA than of an alternative CoA thioester. Interestingly, not all tested potential acceptor acids could serve as a substrate. In particular, acetate that should be small enough to occupy the active site cavity was not used by Mct. On the other hand, succinate was accepted in the presence of varying CoA donors. Yet, Mct showed only poor catalytic efficiency (at least 6 orders of magnitude lower than for the intra-molecular CoA transfer) with succinate as the CoA acceptor. Preventing the diffusion of substrates in and out of the active site is likely the reason why the Mct reaction proceeds so fast in comparison to the inter-molecular transfers catalyzed by the other family III CoA transferases.^10,20,34−38^

In conclusion, our data provide detailed molecular insights into the structural and mechanistic differences between intra- and inter-molecular family III CoA transferases, explaining how “corking up” the active site with the CoA substrate allows Mct to achieve excellent selectivity toward its native substrates, efficiently preventing unwanted side reactions.

## Supporting Information Available

Assessment of the purity of used enzymes and CoA esters; demonstration of the stability of mesaconyl-CoA derivates at 55 °C; and list of PFAM IDs for the different CoA transferase families (PDF)

## Accession Codes

PDB-ID for Mct – apo form: 8APR; PDB-ID for Mct – substrate-bound form: 8APQ; Uniprot accession for Mct: A9WC36.
