# Supplementary material for: Structural Basis for a Cork-Up Mechanism of the Intra-Molecular Mesaconyl-CoA Transferase
Source: Biochemistry. 2022 Dec 19;62(1):75–84. doi: 10.1021/acs.biochem.2c00532 (PMC9813914; doi:10.1021/acs.biochem.2c00532)
Supplement: Supplementary file 1 — bi2c00532_si_001.pdf [file bi2c00532_si_001.pdf]

Supporting Information for "Structural basis for a cork-up mechanism of the intra-molecular mesaconyl-CoA transferase"

Pascal Pfister[1], Jan Zarzycki[1], Tobias J. Erb[1,2]*

[1] Department of Biochemistry & Synthetic Metabolism, Max Planck Institute for Terrestrial Microbiology, Karl-von-Frisch Str. 10, 35043 Marburg, Germany

[2] SYNMIKRO Center for Synthetic Microbiology, Karl-von-Frisch Str., 14, 35032 Marburg, Germany

*corresponding author: toerb@mpi-marburg.mpg.de

Supporting results

Reliability of mesaconyl-CoA separation

Stored samples of mesaconyl-CoA were tested for their purity via HPLC-MS as described in the methods section.

The sample of pooled mesaconyl-C1-CoA contained less than 0.3% of mesaconyl-C4-CoA judged by relative ion count (Fig. S1A). The sample of mesaconyl-C4-CoA contained no detectable contamination of mesaconyl-C1-CoA judged by relative ion count.

Mesaconyl-CoA hydrolysis

Mesaconyl-CoA derivatives were incubated in previous described assay matrix (300 µL containing 500 µM CoA, 200 mM HEPES, $pH_{55°C}$ 7.5) at 55°C for 180 hours. Mesaconyl-C1-CoA was additionally incubated in presence of 3 µg Mct. At different time points, CoA hydrolysis was monitored by measuring the remaining absorbance at 290 nm. Samples were checked for alternative byproducts via HPLC-MS. The only detectable products were free CoA or dimerized CoA.

The hydrolysis half-lives (Fig. S1B) were longer than 30 h and 14 h for mesaconyl-C1-CoA and mesaconyl-C4-CoA, respectively. When mesaconyl-C1-CoA was incubated with Mct, the hydrolysis half-life was similar to that of mesaconyl-C4-CoA (about 12 h), within the margin of error. Here, Mct produced mesaconyl-C4-CoA, which then hydrolyzes faster than mesaconyl-C1-CoA alone. The uniform hydrolysis of mesaconyl-CoA in the presence of Mct with similar rates as mesaconyl-C4-CoA alone suggests that Mct remained active throughout the whole experiment. Judging from these results, the effect of mesaconyl-CoA instability on our enzymatic assays was negligible.

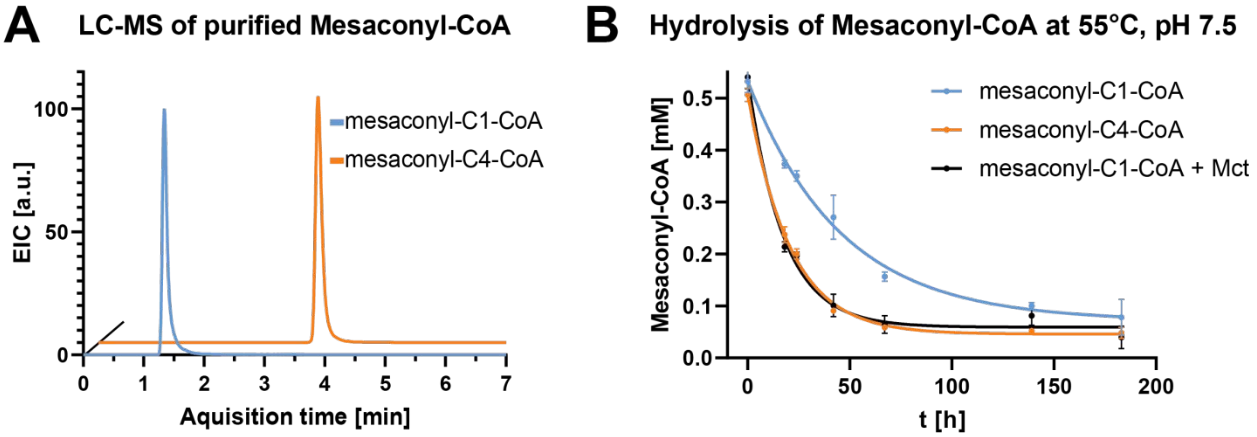

	mes-C1-CoA	mes-C4-CoA	mes-C1-CoA + Mct
Half Life [h]	32 ± 6	14 ± 1	12 ± 2

Figure S1. Purity and stability of mesaconyl-CoAs. *(A) HPLC-MS EIC chromatograms of purified mesaconyl-C1-CoA (blue) and mesaconyl-C4-CoA (orange). (B) Mesaconyl-CoA hydrolysis over time. Mesaconyl-C1-CoA alone (blue) has a 2-fold higher half-life then mesaconyl-C4-CoA (orange) and mesaconyl-C1-CoA incubated with Mct (black) at 55 °C pH 7.5. "±" indicates the 95 % confidence interval.*

Enzyme purity

Protein purity was assessed by SDS-PAGE. Stored samples of the enzyme after Ni-NTA affinity chromatography, as well as after anion exchange chromatography were boiled in 4× SDS-loading buffer (0.2 M Tris-HCl, 0.4 M DTT, 277 mM 8.0% (w/v) SDS, 6 mM Bromphenol blue, 4.3 M glycerol) for 5 minutes at 100 °C. After staining the gel for 10 minutes with GelCode™ Blue Safe staining solution (Thermo Fisher, USA) it was destained with ddH_2O.

After Ni-NTA affinity chromatography, a band corresponding to the size of Mct was already dominant on the gel. A few bands not corresponding to Mct could be removed by anion exchange chromatography using Q-sepharose.

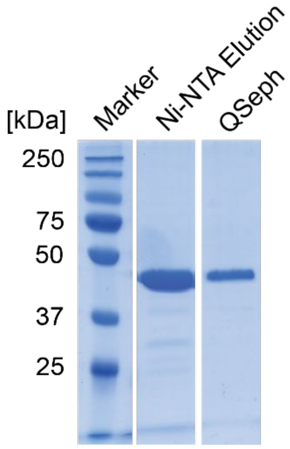

Figure S2. SDS-PAGE results of purification steps of Mct. *Precision Plus Protein Dual Color (Bio-rad, USA) was used as marker (left lane) for size determination. Enriched Mct (10 µg) after Ni-NTA affinity purification (middle lane) already shows as dominant band at ~45 kDa. After the anion exchange (right lane, 6 µg), the only visible band left is at around 45 kDa. Mct has a calculated mass of 44.8 kDa per monomer.*

Supporting tables

Table S1. Nomenclature and PFAM IDs for the different CoA transferase families.

**CoA-transferase family***	**Canonical nomenclature**	**Pfam**
Cat1	Family I	13336, 02550
OXCT1	Family I	01144
Gct	Family I	01144
CitF	Family II	04223
McdA	Family II	16957
Frc	Family III	02515

*Proposed classification according to Hackmann, 2022.
